# Pancreatic Head Cancer Masquerading as Distal Cholangiocarcinoma: Diagnostic Challenges, Tumor Characteristics, and Oncologic Outcomes

**DOI:** 10.3390/cancers18050870

**Published:** 2026-03-08

**Authors:** Kenta Aso, Ryuji Yoshioka, Atsushi Takahashi, Shoichi Irie, Yoshinori Takeda, Yoshihiro Hirata, Takaaki Kato, Hirofumi Ichida, Yoshihito Kotera, Yoshihiro Mise, Yuki Fukumura, Akio Saiura

**Affiliations:** 1 Department of Hepatobiliary-Pancreatic Surgery, Juntendo University School of Medicine, 2-1-1 Hongo, Bunkyo-ku, Tokyo 113-8421, Japan; k.aso.xy@juntendo.ac.jp (K.A.); a-takahashi@juntendo.ac.jp (A.T.); s.irie.fo@juntendo.ac.jp (S.I.); yo-takeda@juntendo.ac.jp (Y.T.); y.hirata.ok@juntendo.ac.jp (Y.H.); t.kato.tb@juntendo.ac.jp (T.K.); hichida@juntendo.ac.jp (H.I.); y.kotera.rs@juntendo.ac.jp (Y.K.); y.mise.xb@juntendo.ac.jp (Y.M.); a-saiura@juntendo.ac.jp (A.S.); 2 Department of Human Pathology, Juntendo University School of Medicine, 2-1-1 Hongo, Bunkyo-ku, Tokyo 113-8421, Japan; yfuku@juntendo.ac.jp

**Keywords:** pancreatic head cancer, pancreatic ductal adenocarcinoma, distal cholangiocarcinoma, preoperative diagnosis, surgical outcomes

## Abstract

**Background/Objectives**: Differentiating pancreatic head cancer (PHC) from distal cholangiocarcinoma (dCCA) remains clinically challenging and directly influences treatment strategy. This study evaluates the clinicopathologic features and outcomes of patients with PHC who were preoperatively designated as dCCA. **Methods**: We retrospectively analyzed patients undergoing pancreatoduodenectomy for suspected dCCA or PHC from 2019 to 2023. Patients were stratified by pre- and postoperative diagnoses into three groups: confirmed dCCA (B-B), confirmed PHC (P-P), and dCCA reclassified as PHC (B-P). Clinicopathologic features, perioperative outcomes, and survival were compared. **Results**: This analysis included 159 patients, B-B = 31, P-P = 115, and B-P = 13. Despite a more advanced stage, a lower R0 rate (*p* = 0.043), and unplanned portal vein resection confined to B-P (*p* < 0.001), overall and recurrence-free survival were comparable to P-P (*p* = 0.363 and 0.183). In multivariable Cox analysis, B-P remained an independent favorable prognostic factor for overall survival (Hazard ratio 0.137, *p* = 0.020). **Conclusions**: Approximately one-third of cases initially diagnosed as dCCA were ultimately PHC. These PHC cases mimicking dCCA demonstrated comparable or even superior survival, suggesting a biologically indolent subset. Refinement of diagnostic criteria and integrated clinicopathologic assessment are essential for optimizing preoperative management strategies.

## Simple Summary

Accurately distinguishing between pancreatic head cancer and distal cholangiocarcinoma before surgery is difficult because they occur in the same area and share similar symptoms. This distinction is critical because treatment plans differ, especially regarding the use of neoadjuvant chemotherapy before surgery. Our study analyzed patients who were initially thought to have distal cholangiocarcinoma but were later diagnosed with pancreatic head cancer after surgery. We found that although these cases often required more complex surgery, the patients had comparatively better survival outcomes than those typical for pancreatic head cancer. This suggests that pancreatic cancers that mimic distal cholangiocarcinoma might be a biologically less aggressive subtype. These findings highlight the need for better diagnostic methods to ensure each patient receives the most effective treatment plan from the start, potentially improving survival for this specific group.

## 1. Introduction

Accurately differentiating pancreatic head cancer (PHC) from distal cholangiocarcinoma (dCCA) poses a persistent challenge in clinical practice. These tumors are anatomically contiguous and frequently share nonspecific symptoms and overlapping radiologic features, while reliable tumor-specific biomarkers remain unavailable [1]. As a result, the preoperative distinction between the two entities is often difficult, even for experienced clinicians. Diagnosis typically relies on a combination of clinical findings, laboratory data, and imaging modalities, including contrast-enhanced computed tomography (CE-CT), magnetic resonance imaging, and endoscopic ultrasound (EUS). However, most prior investigations have focused on the diagnostic utility of these modalities in staging or assessing resectability rather than on their capacity to differentiate PHC from dCCA before surgery [2,3,4,5,6,7].

Despite the frequency of this diagnostic dilemma, few studies have systematically compared patients preoperatively diagnosed with dCCA who were ultimately found to have pancreatic ductal adenocarcinoma (PDAC) on final pathology, in contrast to those accurately diagnosed with PDAC both pre- and postoperatively [8,9]. In particular, such comparative studies from high-volume institutions in Japan remain highly limited. Clarifying these distinctions’ clinical, surgical, and oncologic implications may help improve preoperative diagnostic strategies and optimize treatment selection. In Japan, neoadjuvant chemotherapy (NAC) is the standard treatment strategy for pancreatic cancer, even in resectable cases [10]. By contrast, dCCA is managed with upfront surgery followed by adjuvant S-1 therapy [11]. These divergent treatment strategies mean that an incorrect preoperative diagnosis may substantially affect long-term postoperative outcomes. The aim of this study was to elucidate real-world data from a Japanese high-volume center regarding the diagnostic accuracy of preoperative differentiation between dCCA and PHC, and to investigate whether discrepancies in preoperative diagnosis influence the clinicopathological characteristics and oncologic outcomes.

## 2. Materials and Methods

### 2.1. Patients

This study included consecutive patients who were preoperatively diagnosed with PHC, or dCCA, and who underwent either open or robot-assisted pancreatoduodenectomy at Juntendo University Hospital between January 2019 and December 2023. Patients with a final pathological diagnosis of noninvasive intraductal papillary mucinous carcinoma (IPMC) and those who underwent conversion surgery for unresectable PHC were excluded. We retrospectively reviewed medical records retrieved from a prospectively maintained database, including baseline characteristics, demographic data, preoperative imaging findings, surgical procedures and details, histopathological findings, postoperative outcomes, and prognoses. This study was conducted in accordance with the ethical principles of the Declaration of Helsinki and was approved by the Institutional Review Board of Juntendo University Hospital (approval number: E25-0209).

### 2.2. Treatment Strategy

NAC was administered to patients who were assessed as having PHC based on preoperative imaging. For patients with resectable disease, the NAC regimen consisted of two cycles of gemcitabine plus S-1, in accordance with the Prep-02/JSAP05 trial [10,12]. For patients with borderline resectable tumors, gemcitabine plus nab-paclitaxel or modified FOLFIRINOX, comprising oxaliplatin, irinotecan, fluorouracil, and leucovorin, were administered [13]. Following NAC, tumor resectability was reassessed using CE-CT, and radical resection was performed in appropriate candidates. In contrast, patients who were preoperatively diagnosed with dCCA did not routinely receive NAC. Therefore, these patients proceeded directly to surgery without preoperative systemic treatment. Postoperative adjuvant chemotherapy was administered using S-1 in accordance with the ASCOT trial [11,14]. During the follow-up period, tumor markers (carcinoembryonic antigen, carbohydrate antigen 19-9 [CA19-9], or Duke pancreatic monoclonal antigen type 2) and imaging studies with CE-CT were performed every 3 months for the first 3 years and every 6 months thereafter up to 5 years.

### 2.3. Surgery

All patients in this study underwent pancreatoduodenectomy, primarily via an open approach. Subtotal stomach-preserving pancreatoduodenectomy was the standard procedure performed. In selected cases diagnosed with invasive IPMC or dCCA preoperatively, robot-assisted pylorus-preserving pancreatoduodenectomy was also performed. The choice of surgical approach was based on the preoperative diagnosis, tumor characteristics, and the general condition of the patient. Conventional lymph node dissection was performed in all cases, following standard oncological principles without excess or omission. In PHC patients where tumor attachment or involvement to portal vein were observed in CT before chemotherapy, portal vein resection (PVR) was performed as previously reported [15,16].

### 2.4. Image Analysis

Preoperative imaging evaluation was routinely performed using CE-CT and EUS. EUS was performed using standard echoendoscopes under conscious sedation, and contrast agents were administered when considered appropriate. When PHC was suspected preoperatively, we generally obtained a histological diagnosis by EUS-guided fine-needle aspiration (FNA) prior to NAC. In contrast, when dCCA was suspected, we prioritized tissue diagnosis (or cytology) during endoscopic retrograde cholangiopancreatography (ERCP) over histological confirmation by EUS-FNA. If vascular invasion within the hepatoduodenal ligament was suspected, intraductal ultrasonography was performed. Rapid on-site evaluation was not available for these procedures during this study period.

**Figure 1:**
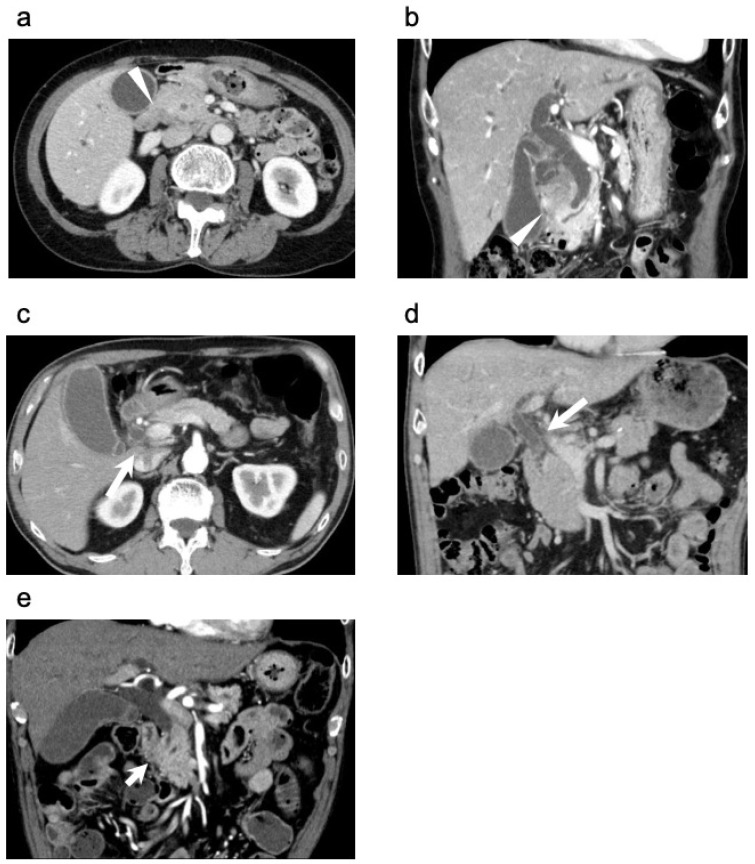
Preoperative contrast-enhanced computed tomography findings in a representative patient initially diagnosed with distal cholangiocarcinoma: (**a**) Axial view showing a lesion extending beyond the bile duct (arrowhead). (**b**) Coronal view also showing extension of the lesion beyond the bile duct (arrowhead). (**c**) Axial view revealing soft tissue density along the hepatoduodenal ligament (large arrow). (**d**) Coronal view confirming soft tissue density extending longitudinally along the hepatoduodenal ligament (large arrow). (**e**) Axial view showing obstruction of the main pancreatic duct (small arrow).

The diagnosis of either dCCA or pancreatic cancer was made by a multidisciplinary team based on preoperative imaging findings, clinical presentation and assessment. In this study, a preoperative diagnosis of dCCA was defined as a lesion primarily located within the bile duct, without obvious evidence of pancreatic involvement. Conversely, PHC was suspected when the lesion was considered to originate from the pancreatic parenchyma or the peripancreatic region, even in cases where no distinct mass was visualized in the pancreatic head. Several imaging features on dynamic CT were considered suggestive of PHC. These included cases in which the main lesion appeared to extend beyond the bile duct (Figure 1a,b), lesions with prominent lateral growth, particularly those spreading along the hepatoduodenal ligament (Figure 1c,d); obstruction of the main pancreatic duct (Figure 1e); and clear signs of local invasiveness into surrounding structures [7,17,18,19]. Bile duct wall thickening, although frequently observed, was not used as a discriminating feature between the two disease entities. Preoperative imaging findings were independently reviewed by two board-certified radiologists, including one with subspecialty expertise in hepatopancreatobiliary imaging, as well as at least two hepatopancreatobiliary surgeons. In cases of disagreement, a consensus was reached through discussion.

### 2.5. Pathological Evaluation

**Figure 2:**
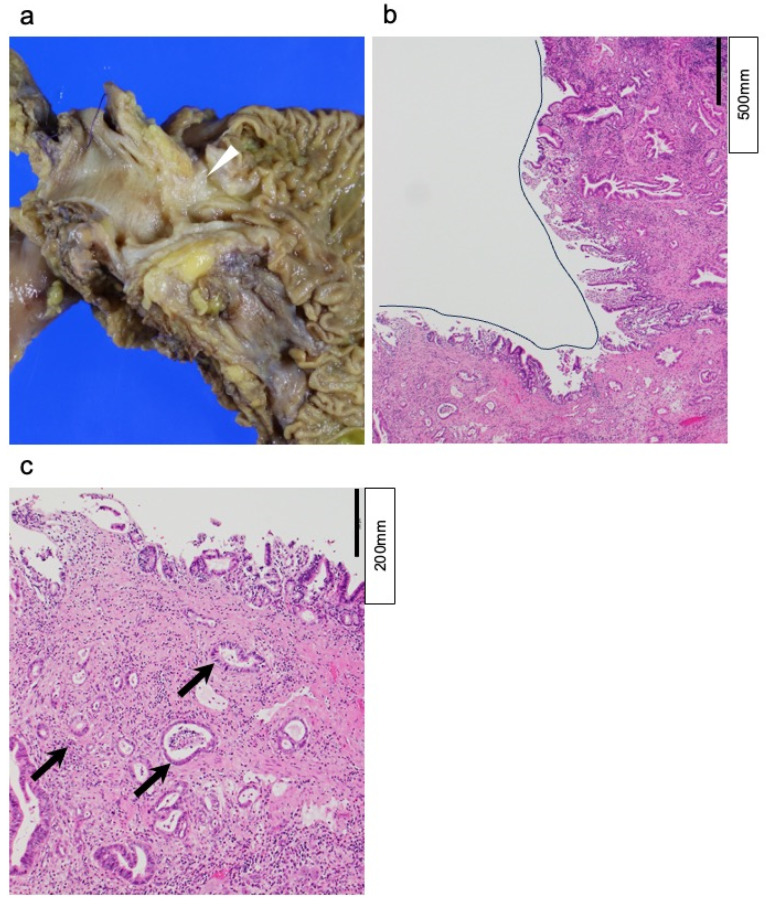
Histopathological findings: (**a**) Macroscopic appearance of the en-bloc resected specimen. The bile duct and pancreatic duct are exposed, and the tumor was identified (arrowhead). (**b**) Hematoxylin and eosin (H&E) staining of the specimen at ×20 magnification revealed the distal bile duct (black line); (**c**) H&E staining of the specimen at ×100 magnification demonstrated infiltration of pancreatic ductal adenocarcinoma into the bile duct wall (arrow).

Pathological findings were independently assessed by two or more board-certified pathologists specializing in HBP diseases at our institution. Specimen processing and diagnostic evaluation were performed in accordance with the General Rules for the Study of Pancreatic Cancer, eighth edition (Japan Pancreas Society) [20], and the General Rules for Clinical and Pathological Studies on Cancer of the Biliary Tract, seventh edition (Japan Biliary Association) [21]. Briefly, the pancreas and the bile duct specimens were cut at 5 mm intervals, and all were processed for pathologic examination. The final diagnosis was determined by consensus following a comprehensive review of histological findings [22]. The pathologists presented a mapped overlay of tumor invasion on photographs of the macroscopic cut surfaces. Tumors were classified as dCCA when (1) carcinoma in situ or high-grade intraepithelial neoplasm was observed at bile duct mucosa, (2) invasive carcinoma involved bile duct wall circumferentially for at least one section, (3) no high-grade intraepithelial neoplasm was observed at pancreatic ducts, (4) tumor progression along the long axis of the bile duct (>2 continuous sections) was observed, and (5) no cancerous involvement of duodenal ampullary region was detected. In contrast, those were classified as PHC when (1) no carcinoma in situ or high-grade intraepithelial neoplasm was present, whereas benign bile duct epithelia were lining the bile duct mucosa (Figure 2). When cases did not satisfy either of the dCCA and PHC criteria, as written above, they were excluded from this study. The histological grade of ductal adenocarcinoma was determined according to the current WHO classification, which is based on combined assessment of glandular differentiation, mucin production, mitotic activity, and nuclear features. In cases with intratumoral heterogeneity, the higher grade was assigned, even if the high-grade component constituted less than 50% of the tumor [23,24].

### 2.6. Statistical Analysis

Overall survival (OS) was defined as the time from the date of diagnosis, based on initial radiological or pathological evaluation, to the date of death from any cause. Recurrence-free survival (RFS) was defined as the time from the date of surgery to the date of first documented recurrence or death. Continuous variables with a normal distribution were compared using the independent Student’s t-test and are presented as means with standard deviations. For variables that did not follow a normal distribution, the Mann–Whitney U test was applied, with results expressed as medians and interquartile ranges. Multivariable analysis was performed using Cox proportional hazards regression with stepwise variable selection. OS and RFS were initially evaluated using the log-rank test, followed by univariate Cox proportional hazards regression analysis to estimate hazard ratios (HRs) and 95% confidence intervals (CIs). Statistical analyses were performed using R (version 4.3.2 for macOS; R Foundation for Statistical Computing, Vienna, Austria) and JMP^®^ Pro 18.1.2 (SAS Institute Inc., Cary, NC, USA).

## 3. Results

**Figure 3:**
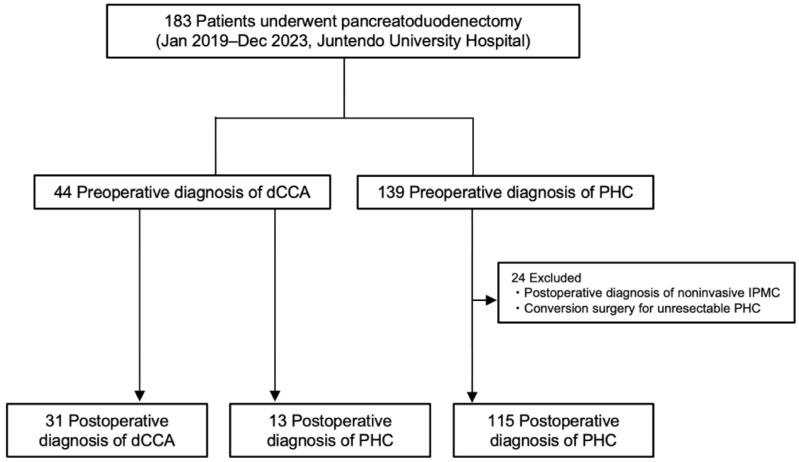
Schematic flowchart of this study: dCCA: distal cholangiocarcinoma; IPMC: intraductal papillary-mucinous carcinoma; PHC: pancreatic head cancer.

A total of 183 patients underwent pancreatoduodenectomy for PHC or dCCA between January 2019 and December 2023. Twenty-four were excluded (postoperative diagnosis of noninvasive IPMC and conversion surgery for initially unresectable PHC), leaving 159 patients for analysis (Figure 3). Preoperatively, 44 were assigned as dCCA and 139 as PHC. The patient baseline characteristics according to pre- and postoperative diagnoses in three groups are shown in Table 1.

### 3.1. Preoperative Comparison Between Three Groups

Among the 44 patients preoperatively diagnosed with dCCA, 31 were confirmed postoperatively as dCCA (B-B group) and 13 as PDAC (B-P group). In addition, of the 128 patients with postoperative PHC, 115 were preoperatively concordant (P-P group) and 13 were initially assigned as dCCA (B-P group). The proportion of diagnostic discrepancy between prior and after surgery in patients with preoperative dCCA was 29.5%. Conversely, there were no cases in which a preoperative diagnosis of PDAC was revised to a postoperative diagnosis of dCCA. The median age was 74 years in the B-P group and 80 years in the B-B group (*p* = 0.104). Preoperative biliary drainage was performed in all B-P patients and in 90.3% of B-B patients (*p* = 0.138). The median total bilirubin level was slightly lower in the B-P group (12 μmol/L) than in the B-B group (17 μmol/L, *p* = 0.149). The main pancreatic duct diameter was significantly larger in the B-P group (2.8 mm vs. 2.1 mm, *p* = 0.038), and portal vein contact was more frequent (46.1% vs. 6.4%, *p* = 0.002). No significant differences were observed in other preoperative variables. Between the P-P and B-P cohorts, B-P patients were significantly older (*p* = 0.036), were more likely to have received preoperative biliary drainage (*p* < 0.001) and had higher amylase (*p* = 0.020) and lower C reactive protein levels (*p* = 0.001). In contrast, the P-P group showed a larger main pancreatic duct diameter (*p* < 0.001), a higher rate of portal vein contact (*p* < 0.001), and more frequent NAC (*p* < 0.001). No other preoperative variables differed significantly.

### 3.2. Postoperative Comparison Between Two Groups with Postoperative Diagnosis of PHC

Detailed postoperative comparisons are summarized in Table 2. The maximum tumor diameter was larger in B-P (38 vs. 25 mm, *p* = 0.009). The lymph node ratio was higher (0.142 vs. 0, *p* = 0.006), the number of metastatic lymph nodes was greater (median 4 vs. 0, *p* = 0.003), and lymph node metastasis was more frequent (76.9% vs. 46.9%, *p* = 0.036). Station 12b lymph node metastasis occurred more often in the B-P (38.4% vs. 3.5%, *p* < 0.001). Bile-duct invasion was present in 100% vs. 41.2% (*p* < 0.001). Soft pancreatic texture was more common in the B-P (76.9% vs. 28.7%, *p* < 0.001). R0 resection was less frequently achieved in B-P (61.5% vs. 84.3%, *p* = 0.043). Portal vein resection was performed in 46.1% vs. 59.1% (*p* = 0.369), while unplanned portal vein resection was conducted only in the B-P (30.7% vs. 0%, *p* < 0.001). Operative time, intraoperative blood loss, 90-day mortality, severe complications (Clavien–Dindo ≥ IIIa), and adjuvant chemotherapy were comparable.

### 3.3. Long-Term Outcomes

Recurrence patterns differed significantly between the two groups (*p* = 0.013). Local recurrence was observed more frequently in the B-P group (30.7% vs. 10.5%), while distant metastases occurred only in the P-P group (38.6% vs. 0%). No lymph node recurrences were reported in either group (Table 3).

**Figure 4:**
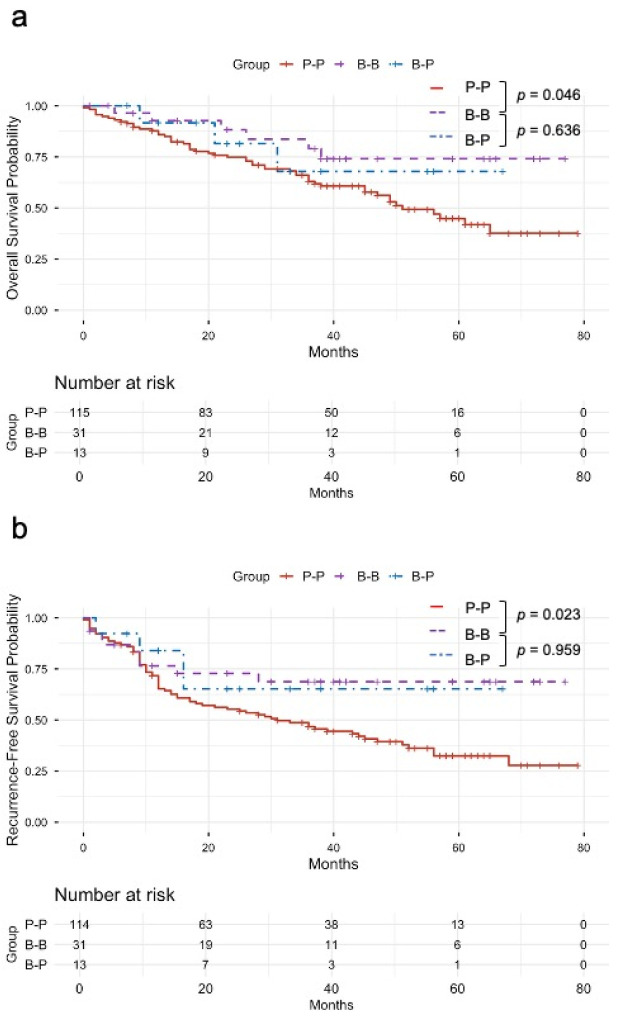
Survival outcomes stratified by diagnostic concordance between initial assessment and definitive pathology: (**a**) Kaplan–Meier curves of overall survival by diagnostic group: P-P, B-B, and B-P groups. (**b**) Kaplan–Meier curves of recurrence-free survival by the same diagnostic groups; Diagnostic groups: P-P = pancreatic head cancer (PHC) on initial assessment and PHC on definitive pathology; B-B = distal cholangiocarcinoma (dCCA) on initial assessment and dCCA on definitive pathology; B-P = dCCA on initial assessment and PHC on definitive pathology.

OS did not differ between the P-P and B-P groups (*p* = 0.363; Figure 4a). 5-year OS was 44.8% in the P-P and 67.9% in the B-P; median OS was 51 months in the P-P group and not reached in B-P group. RFS was also comparable (*p* = 0.183; Figure 4b). 1- and 3-year RFS were 65.3% and 46.7% in the P-P, and 83.9% and 65.3% in the B-P group. In multivariable Cox analysis for OS, the B-P group was associated with a lower risk of death than the P-P (HR 0.137, 95% CI 0.026–0.735, *p* = 0.020). Additional independent predictors of worse OS were higher CA19-9 (HR 1.18, *p* = 0.015), larger tumor size (HR 1.03, *p* = 0.024), and higher lymph node ratio (HR 2.10, *p* < 0.001), while older age was associated with a lower risk of death (HR 0.965, *p* = 0.001). For RFS, B-P showed a favorable but non-significant association (HR 0.228, 95% CI 0.046–1.118, *p* = 0.068). Independent predictors of shorter RFS were higher CA19-9 (HR 1.20, *p* = 0.006) and higher lymph node ratio (HR 1.62, *p* = 0.004), whereas older age was associated with a lower risk of recurrence (HR 0.966, *p* < 0.001).

## 4. Discussion

This study demonstrated the persistent difficulty in achieving accurate preoperative differentiation between dCCA and PHC, highlighting that a subset of PHC remains indistinguishable from dCCA before surgery (B-P group). We compared the B-B with the B-P groups preoperatively and the P-P with the B-P groups postoperatively. We demonstrated that, despite incorporating multidisciplinary imaging assessment and established diagnostic criteria, preoperative identification of B-P cases remains inherently challenging, with a subset inevitably exhibiting pre- to postoperative diagnostic discordance. Notably, the B-P group underwent upfront surgery without NAC and exhibited a higher burden of lymph node metastases yet achieved OS and RFS rates comparable to the P-P group. This paradox, more advanced nodal disease but equivalent prognosis, together with significant differences in histological grade between groups, suggests that distinct tumor biology may underlie the observed outcomes. Furthermore, in multivariable analysis for OS, the B-P group remained an independent prognostic factor associated with a lower risk of death. By integrating detailed preoperative imaging analysis, comprehensive pathological mapping, and survival assessment within a single high-volume institutional cohort, our findings address a persistent diagnostic blind spot in HPB oncology and open new avenues for refining preoperative risk stratification and treatment selection.

Differentiating between PDAC and dCCA, especially in periampullary tumors, remains challenging, with approximately 16% of cases ultimately reclassified at definitive pathology [9,18,25]. Both entities share overlapping clinical features and imaging characteristics [7]. When stratified by disease type, the accuracy of preoperative diagnosis was 84% for pancreatic cancer, 71% for dCCA, 73% for ampullary cancer, and 73% for duodenal cancer [9]. These figures highlight the particular diagnostic challenge associated with dCCA. Notably, despite a standardized preoperative diagnostic algorithm combining CE-CT and EUS, with ERCP or magnetic resonance imaging as indicated, we observed no diagnostic discordance among cases preoperatively assigned as PHC. This underscores the high specificity and reliability of our preoperative assessment, particularly when compared to previous reports of a 12–13% misdiagnosis rate in this direction [26]. In contrast, approximately 30% of patients assigned preoperatively as dCCA were postoperatively confirmed as PHC. Despite the integration of multiple diagnostic modalities, including CE-CT, EUS, and intraductal ultrasonography, preoperative differentiation between dCCA and PHC remains inherently challenging. This diagnostic dilemma persists because both tumors originate in close proximity within the pancreatic head and often share overlapping imaging characteristics. In the comparison of preoperative factors between the B-P and B-B cohorts, main pancreatic duct diameter differed statistically (median 2.8 vs. 2.1 mm, *p* = 0.038), although the absolute difference was small, which might indicate limited discriminatory value of preoperative imaging for distinguishing PHC from dCCA. To overcome these diagnostic hurdles, meticulous diagnostic EUS and thorough EUS-guided tissue sampling might be instrumental, particularly for cases of dCCA diagnosed on preoperative CE-CT [27,28]. Clinical consideration of these diagnostic approaches may be required, as accurate preoperative diagnosis has significant clinical implications.

Treatment strategies for PHC and dCCA differ substantially. For PDAC, NAC is now regarded as a standard of treatment as endorsed by major international guidelines [29], even for resectable status [20]. NAC improves the likelihood of achieving R0 resection and allows early treatment of micrometastases [30,31]. Furthermore, surgical planning is influenced by whether the tumor is PHC or dCCA. When PHC is suspected preoperatively and radiologic findings suggest portal vein contact or involvement, PVR can be proactively incorporated into the surgical strategy [15,16]. In such cases, surgeons avoid dissecting near the tumor and instead plan for en-bloc resection, thereby minimizing intraoperative complications and optimizing oncologic outcomes. In addition, perioperative treatment strategies continue to diversify for both pancreatic and biliary tract cancers, accurate preoperative diagnosis has become increasingly critical [32,33,34]. This divergence in treatment approach underscores the importance of correct preoperative diagnosis. Compared with the P-P group, the B-P group exhibited a higher rate of unplanned PVR (30.7% vs. 0%, *p* < 0.001) and a lower R0 rate (61.5% vs. 84.3%, *p* = 0.043), along with a higher local recurrence rate (30.7%). These patterns might reflect the impact of NAC and an en-bloc resection policy that includes PVR.

Reports specifically addressing the preoperative distinction between PHC and dCCA are limited. A diagnostic scoring system incorporating CA19-9 > 230 U/mL, C-reactive protein > 10 mg/dL, and main pancreatic duct dilatation > 3 mm has been proposed, with a score > 1 suggesting PDAC (Area Under the ROC curve 0.74, *p* < 0.001) [18]. In addition, Roessel et al. identified clinical predictors of PDAC in cases initially diagnosed as dCCA, including >10% weight loss, CA19-9 > 160 U/mL, vascular invasion on imaging, tumor size > 20 mm, and positive EUS-guided fine-needle aspiration [9]. Notably, in PDAC cases initially classified as dCCA and subsequently confirmed as PDAC, the median OS was comparable to that of preoperatively concordant PDAC managed without neoadjuvant therapy (21.5 vs. 19.4 months), indicating no evident impact of diagnostic discordance on short-term survival. Relatedly, pancreatic groove cancer, a periampullary tumor that is particularly difficult to differentiate from distal cholangiocarcinoma, has been reported to exhibit well-differentiated histology in 43% of cases, with perineural invasion present in 90% and both lymphovascular invasion and lymph node metastasis identified in 83%. Moreover, well-differentiated tumors have been reported to be associated with better survival outcomes than moderately or poorly differentiated tumors [8].

Molecular subtype classification of PDAC, particularly the Moffitt classification, may partly explain these findings. This system stratifies tumors into Classical and Basal-like subtypes, with the Basal-like subtype associated with poor prognosis [35,36,37]. The Classical subtype shows higher tumor differentiation, consistent with the significantly better differentiation observed in the B-P group than in the P-P group. Although molecular subtyping was not performed, subtype-related tumor biology might have contributed to the observed prognostic differences. These observations suggest that the B-P group might represent a biologically distinct subgroup of pancreatic cancer with a less aggressive clinical course. While these biological characteristics could mitigate the impact of unfavorable surgical results, future large-scale studies are necessary to fully elucidate the relationship between tumor biology and diagnostic discordance in these patients.

This study has several limitations. First, it is a retrospective single-center study, subject to inherent biases in patient selection and data collection. Second, the total number of patients with a preoperative diagnosis of dCCA was small (n = 44), and specifically, the B-P group consisted of only 13 patients. This small sample size, especially when compared with the P-P group (n = 115), represents a major limitation in achieving robust statistical comparisons and may have introduced potential biases. Third, although two pre-dCCA patients received preoperative systemic treatment for suspected advanced disease, these were not standardized protocols. Fourth, our pathological classification of P-P and B-P is histology-based, and not molecular-based. Molecular or genomic characteristics could further clarify tumor origin or subtype classification. Fifth, 29.5% of patients who were preoperatively diagnosed with dCCA were ultimately found to have PDAC, suggesting a substantial rate of diagnostic discrepancy. One contributing factor may be the tendency to classify tumors with extensive longitudinal spread along the bile duct as cholangiocarcinoma with extensive biliary involvement, which could have reduced the number of cases specifically diagnosed as dCCA. Lastly, the substantial difference in neoadjuvant chemotherapy use between groups (67.8% vs. 15.3%) may have introduced residual confounding that could have influenced survival outcomes. Therefore, the present findings should be considered hypothesis-generating and require validation in larger, multi-institutional cohorts.

## 5. Conclusions

In conclusion, a proportion of cases assigned as dCCA preoperatively may represent PDAC. PDAC presenting with a dCCA-like phenotype may constitute a biologically more indolent subset, and in our cohort, this pre- to postoperative diagnostic discordance was not associated with inferior survival. Complete preoperative discrimination remains difficult, underscoring the need for refined diagnostic pathways and integrated clinicopathological assessment to optimize treatment selection.

## Figures and Tables

**Table 1:** Preoperative demographic characteristics of patients according to pre- and postoperative diagnoses in three groups.

Variables	P–P (n = 115)	B-B Group (n = 31)	B-P Group (n = 13)	*p* Value B-B vs. B-P	*p* Value P-P vs. B-P
Preoperative findings					
Age, years	69 (59–86)	80 (58–88)	74 (70.5–77.5)	0.104	0.036
Sex, male,	61 (53.0)	28 (90.3)	7 (53.8)	0.006	0.956
BMI, kg/m^2^	21.6 (13.2–33.0)	22.8 (18.8–28.6)	21.1 (17.1–23.5)	0.004	0.098
Preoperative lab					
Total bilirubin, μmol/L	10.8 (2.7–120.0)	18.6 (8.6–239.4)	13.0 (7.0–25.7)	0.149	0.066
Albumin, g/L	38 (26–48)	37 (26–45)	38 (28–40)	0.716	0.250
CRP, mg/L	1.1 (0.2–111)	5 (0.3–119)	0.6 (3.5–44.7)	0.816	0.001
Amylase, U/L	82 (22–313)	124 (67–540)	179 (140–243)	0.037	0.020
HbA1c, mmol/mol	43 (30–104)	39 (20–78)	40 (26–71)	0.846	0.147
CEA, µg/L	3.0 (0.5–27)	2.1 (0.6–40.3)	1.8 (1.2–19.9)	0.661	0.084
CA19-9, U/mL	29 (1.3–1288)	21 (7–730)	34 (9–1455)	0.193	0.310
MPD diameter, mm	5.7 (1.1–18)	2.1 (1.0–11.1)	2.8 (1.4–4.9)	0.038	<0.001
Contact with the PV	68 (59.1)	2 (6.45)	6 (46.1)	0.002	<0.001
Preoperative biliary drainage	45 (39.1)	28 (90.3)	13 (100)	0.138	<0.001
Neoadjuvant chemotherapy	78 (67.8)	3 (9.6)	2 (15.3)	0.586	<0.001

Data are number (percentage), median (range). Abbreviations: B-B, concordant diagnosis of distal cholangiocarcinoma; BMI, body mass index; B-P, preoperatively diagnosed as distal cholangiocarcinoma and postoperatively confirmed as pancreatic head cancer; CA19-9, carbohydrate antigen 19-9; CEA, carcinoembryonic antigen; HbA1c, glycated hemoglobin; MPD, main pancreatic duct; P-P, concordant diagnosis of pancreatic head cancer.

**Table 2:** Surgical outcomes and postoperative pathological findings in patients with postoperative diagnosis of pancreatic head cancer in two groups.

Variables	P-P Group (n = 115)	B-P Group (n = 13)	*p* Value
Surgical procedures			
Portal vein resection	68 (59.1)	6 (46.1)	0.369
Arterial resection	2 (1.7)	1 (7.6)	0.178
Unplanned portal vein resection	0 (0)	4 (30.7)	<0.001
Pancreatic texture, hard	82 (71.3)	3 (23.0)	<0.001
Operation time, min	488 (245–989)	513 (353–717)	0.450
Blood loss, mL	218 (15–1100)	250 (20–650)	0.770
Complications, C–D grade ≥ IIIa	17 (14.7)	2 (15.3)	0.953
90-day mortality	2 (1.7)	0 (0)	0.511
Adjuvant chemotherapy	91 (79.1)	12 (92.3)	0.211
Tumor size, maximum, mm	25 (3–65)	38 (20–60)	0.009
Lymph node metastasis			
Positive	54 (46.9)	10 (76.9)	0.035
LNR	0 (0–0.368)	0.142 (0–0.411)	<0.001
MPD invasion	16 (13.9)	1 (7.6)	0.751
Bile duct invasion	47 (41.2)	13 (100)	<0.001
R0 resection	97 (84.3)	8 (61.5)	0.042
Margin, mm	5 (0–50)	2 (0–10)	0.058
Differentiation			
Well	33 (28.7)	9 (69.2)	0.003
Moderately	39 (33.9)	2 (15.3)	0.150
Poorly	19 (16.5)	0 (0)	0.035
Metastatic lymph node sites			
Station 8	2 (1.7)	1 (7.6)	0.187
Station 12b	4 (3.5)	5 (38.4)	<0.001
Station 13	50 (43.8)	6 (46.1)	0.874
Station 17	14 (12.2)	1 (7.6)	0.608

Data are number (percentage), median (range). Abbreviations: B-P, preoperatively diagnosed as distal cholangiocarcinoma and postoperatively confirmed as pancreatic head cancer; C-D, Clavien-Dindo; LNR, lymph node ratio; MD, median; MPD, main pancreatic duct; P-P, concordant diagnosis of pancreatic head cancer.

**Table 3:** Postoperative recurrence patterns of patients.

Variables	P-P Group (n = 115)	B-B Group (n = 31)	B-P Group (n = 13)	*p* Value
Recurrence pattern				0.013
Local recurrence	12 (10.5)	2 (6.6)	4 (30.7)	
Distant metastasis	44 (38.6)	8 (26.6)	0 (0)	
Lymph node recurrence	1 (0.8)	0 (0)	0 (0)	

Data are number (percentage). Abbreviations: B-B, concordant diagnosis of distal cholangiocarcinoma; B-P, preoperatively diagnosed as distal cholangiocarcinoma and postoperatively confirmed as pancreatic head cancer; P-P, concordant diagnosis of pancreatic head cancer.

## Data Availability

The data presented in this study are available on request from the corresponding author due to patient privacy and ethical restrictions but are available from the corresponding author upon reasonable request.

## Abbreviations

The following abbreviations are used in this manuscript:

CE-CT	Contrast-enhanced computed tomography
CIs	Confidence intervals
dCCA	Distal cholangiocarcinoma
EUS	Endoscopic ultrasound
ERCP	Endoscopic retrograde cholangiopancreatography
FNA	Fine-needle aspiration
IPMC	Intraductal papillary-mucinous carcinoma
HRs	Hazard ratios
NAC	Neoadjuvant chemotherapy
OS	Overall survival
PDAC	Pancreatic ductal adenocarcinoma
PHC	Pancreatic head cancer
PVR	Portal vein resection
RFS	Recurrence-free survival
